# Involvement of the claustrum in the cortico-basal ganglia circuitry: connectional study in the non-human primate

**DOI:** 10.1007/s00429-024-02784-6

**Published:** 2024-04-14

**Authors:** Elena Borra, Gemma Ballestrazzi, Dalila Biancheri, Roberto Caminiti, Giuseppe Luppino

**Affiliations:** 1 Unità di Neuroscienze, Dipartimento di Medicina e Chirurgia, Università di Parma, 43100 Parma, Italy; 2 Neuroscience and Behaviour Laboratory, Istituto Italiano di Tecnologia (IIT), 00161 Rome, Italy

**Keywords:** Claustrum, Striatum, Cortical networks, Macaque

## Abstract

The claustrum is an ancient telencephalic subcortical structure displaying extensive, reciprocal connections with much of the cortex and receiving projections from thalamus, amygdala, and hippocampus. This structure has a general role in modulating cortical excitability and is considered to be engaged in different cognitive and motor functions, such as sensory integration and perceptual binding, salience-guided attention, top-down executive functions, as well as in the control of brain states, such as sleep and its interhemispheric integration. The present study is the first to describe in detail a projection from the claustrum to the striatum in the macaque brain. Based on tracer injections in different striatal regions and in different cortical areas, we observed a rough topography of the claustral connectivity, thanks to which a claustral zone projects to both a specific striatal territory and to cortical areas involved in a network projecting to the same striatal territory. The present data add new elements of complexity of the basal ganglia information processing mode in motor and non-motor functions and provide evidence for an influence of the claustrum on both cortical functional domains and cortico-basal ganglia circuits.

## Introduction

The claustrum is an ancient telencephalic subcortical structure formed by a thin, tortuous sheet of grey matter, extending in rostrocaudal direction, mostly between the insular cortex and the putamen. It is present not only in mammals but also in reptiles (Norimoto et al. 2020), thus allowing the analysis of the preservation and evolution of the structure and potential functions from the brain of a common ancestor of reptiles and mammals. In mammals, the claustrum displays extensive, reciprocal connections with much of the cortex and is a target of thalamic, amygdalar, and hippocampal projections (for review, see Jackson et al. 2020). Specifically, in rodents claustro-cortical connectivity has been described as topographically organized in modality-specific claustral sectors, with primary input originating from limbic, associative, and motor areas and weaker ones from sensory parietal, temporal, or occipital areas. Other studies hold that cortical areas sharing corticocortical connections are reciprocally connected to specific claustral zones. In recent years, the potential role of the claustrum has been discussed by several review articles (e.g., Jackson et al. 2020; Smith et al. 2020; Nikolenko et al. 2021; Atilgan et al. 2022; Madden et al. 2022). Among the possible functions, a role of the claustrum has been suggested in sensory integration and perceptual binding, sensorimotor processing, salience-guided attention, top-down executive functions requiring a frontal cortical control over posterior cortical regions, as well as in sleep regulation.

Recently, in a study focused on the connectivity of white matter neurons, we found that in non-human primates the claustrum is a source of projections to the striatum (Borra et al. 2020), which were reported also by Arikuni and Kubota (1985) and Griggs et al. (2017), but have not been so far described in detail or discussed in models of claustral functions. These projections further enrich the complex pattern of information convergence in the striatum emerging from recent studies. Indeed, there is evidence that interconnected cortical areas display overlapping corticostriatal projections in restricted striatal territories (Gerbella et al. 2016; Choi et al. 2017). Furthermore, the projections from the various cortical areas to a specific striatal zone can differ in their laminar origin (Borra et al. 2021). Finally, in macaques the striatum is target of robust crossed cortical projections originating mostly from contralateral prefrontal, premotor, motor, and rostral cingulate areas, whose amount in some cases tends to equal that from ipsilateral ones (Innocenti et al. 2017; Borra et al. 2022). In humans, such crossed projections stem also from parietal and temporal areas related to language processing (Innocenti et al. 2017), although their role remains to be elucidated. The newly identified claustro-striatal projections could provide the substrate for a more extensive role of the claustrum in coordinating large-scale cortical networks and represent a further factor of complexity of the information processing mode of the basal ganglia in motor and non-motor functions.

In the present study, the source and topography of claustral projections to the motor and pre-commissural putamen and to the caudate head were analyzed and compared with those of the claustral projections to different cortical regions.

## Methods

### Subjects, surgical procedures, and injection sites

In the present study, the claustrostriatal connectivity has been analyzed based on the results from retrograde tracer injections in post-commissural and pre-commissural putamen and in the caudate head in 4 *Macaca mulatta*. As in all these cases the labeled corticostriatal neurons largely involved frontal and parietal areas, the comparison with the claustrocortical connectivity was based on results from retrograde tracer injections in 18 additional macaques (9 *Macaca fascicularis*, 4 *Macaca nemestrina*, and 5 *Macaca mulatta*), mostly in prefrontal, frontal motor, and parietal areas. Table 1 summarizes the cases used in the present study. As most of these cases have been already used in previous studies focused on corticocortical or corticostriatal connectivity (see for references Table 1), in this section for the sake of clarity and consistency we reuse the description of authorizations, surgical procedures, brain perfusion, histological processing, and immunohistochemical procedures already provided in previous studies (Matelli et al. 1998; Luppino et al. 1993, 2001, 2003; Rozzi et al. 2006; Borra et al. 2008, 2010, 2011, 2019, 2022; Gerbella et al. 2010; 2013; Caminiti et al. 2021).

**Table 1:** Cases used in the present study

Case	Species (Macaca)	Sex	Age (y)	Weight (kg)	Hem	Region	Tracer	Amount
Striatum								
71	*Mulatta*	F	6.5	3.3	L	Post-commissural putamen^a^	FB 3%	0.3 µl
75	*Mulatta*	M	6	3.5	R	Post-commissural putamen^a^	CTB-Alexa488 1%	1 µl
					R	Caudate body^a^	DY 2%	0.25 µl
76	*Mulatta*	M	9	15	R	Caudate head^a^	WGA 4%	0.3 µl
77	*Mulatta*	M	9	15	R	Pre-commissural Putamen^a^	WGA 4%	0.2 µl
					L	Post-commissural putamen^a^	CTB-Alexa488 1%	1 µl
Frontal motor and prefrontal areas								
01	*Fascicularis*	F	8	4	L	F6^b^	FB 3%	0.2 µl
					L	F3 arm^b^	DY 2%	0.2 µl
					L	F3 leg^b^	TB 5%	2 × 0.2 µl
11	*Nemestrina*	F	11	5.6	R	F2vr^c^	FB 3%	1 × 0.2 µl. 1 × 0.15 µl
					R	F7 caudal^c^	DY 2%	2 × 0.2 µl
					R	F7 rostral^c^	TB 5%	1 × 0.2 µl. 1 × 0.3 µl
13	*Fascicularis*	F	12	6.7	L	F7^c^	FB 3%;	0.2 µl
					L	F5c	DY 2%	0.4 µl
					L	F1^c^	TB 5%	0.2 µl
20	*Nemestrina*	F	17	6.6	L	F1^d^	CTB-gold 0.5%	1 µl
23	*Fascicularis*	M	17	6.8	L	46v interm^e^	FB 3%	2 × 0.2 µl
					L	46d caudal^f^	DY 2%	2 × 0.3 µl
30	*Nemestrina*	M	20	10	R	F5p	CTB-Alexa488 1%	2 µl
					R	45B^e^	FB 3%	0.2 µl
36	*Fascicularis*	M	8	9	L	45B^e^	FB 3%	0.2 µl
					L	FEF^e^	DY 2%	0.2 µl
43	*Mulatta*	M	8	10	L	12r interm^g^	FB 3%	0.2 µl
52	*Mulatta*	M	11	9	L	46v rostral^h^	LYD 10%	1.3 µl
58	*Fascicularis*	F	9	3	L	9	LYD 10%	1.5 µl
60	*Fascicularis*	M	3.5	3.2	L	46d interm^f^	FB 3%	0.3 µl
					L	46d interm^f^	CTB-Alexa594 1%	2 µl
					L	8r^f^	CTB-Alexa488 1%	1.8 µl
64	*Fascicularis*	F	4	3.1	L	46d rostral^f^	CTB-Alexa488 1%	2 × 0.75 µl
75	*mulatta*	M	6	3.5	R	13	FB 3%	2 × 0.35 µl
	Parietal, temporal, and occipital areas							
20	*nemestrina*	F	17	6.6	L	PEip	FB	0.2 µl
					L	AIP^i^	DY 2%	0.2 µl
					L	V2	TB	0.2 µl
27	*nemestrina*	F	9	5.3	R	PF^j^	FB 3%	0.2 µl
					R	Opt^j^	DY2%	0.2 µl
					R	Opt^j^	TB 5%	0.2 µl
29	*fascicularis*	F	7	3.5	R	PFG^j^	FB 3%	0.2 µl
					R	PF^j^	DY 2%	0.2 µl
					R	PG^j^	TB 5%	0.2 µl
39	*fascicularis*	M	9	8	R	TEa/m	WGA 4%	2 × 0.15 µl
42	*mulatta*	M	7.5	8.5	R	TEa/m	WGA 4%	2 × 0.15 µl
73	*mulatta*	M	9	12.5	L	PEip^k^	FB 3%	0.3 µl
					L	MIP^k^	DY 2%	0.3 µl
Multiple regions								
62	*mulatta*	F	4	4.5	L	46v/12r	CTB-Alexa488 1%	2 × 1.2 µl
					L	F1	FR 10%	2 × 1 µl
					L	AIP/PFG	LYD 10%	3 × 1 µl

Cortical connections described in ^a^Borra et al. (2022), ^b^Luppino et al. (1993), ^c^Matelli et al. (1998), Luppino et al. (2001, 2003), ^d^Borra et al. (2010), ^e^Gerbella et al. (2010), ^f^Borra et al. (2019), ^g^Borra et al. (2011), ^h^Gerbella et al. (2013), ^i^Borra et al. (2008), ^j^Rozzi et al. (2006), ^k^Caminiti et al. (2021)

Animal handling as well as surgical and experimental procedures complied with the European (directives 86/609/EEC, 2003/65/CE, and 2010/63/EU) and Italian (D.L. 116/92 and 26/2014) laws in force on the humane care and use of laboratory animals. All procedures were approved by the Veterinarian Animal Care and Use Committee of the University of Parma and of the University of Rome SAPIENZA and authorized by the Italian Ministry of Health.

Under general anesthesia (see previous studies indicated in Table 1 for the different protocols) and aseptic conditions, each animal was placed in a stereotaxic apparatus and an incision was made in the scalp. The skull was trephined to remove the bone and the dura was opened to expose a small cortical region. After the neural tracer injections, the dura flap was sutured, the bone replaced, and the superficial tissues sutured in layers. During surgery, hydration was maintained with saline, and heart rate, blood pressure, respiratory depth, and body temperature were continuously monitored. Upon recovery from anesthesia, the animals were returned to their home cages and closely observed. Dexamethasone and prophylactic broad-spectrum antibiotics were administered pre- and postoperatively, as were analgesics.

### Tracer injections and histological procedures

**Fig. 1:**
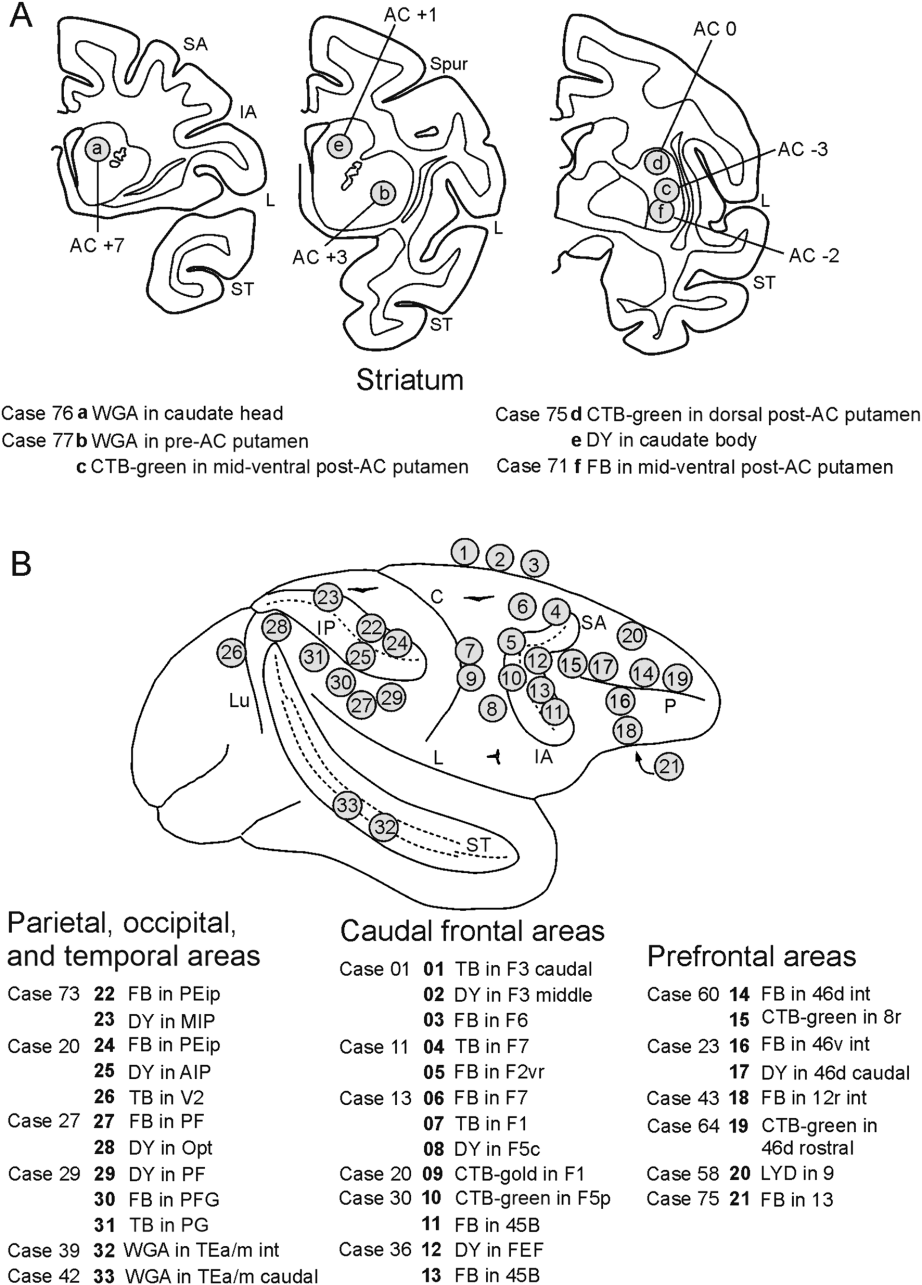
Location of the injection sites in the striatum and the cortex. **A** The location of the injection sites in the putamen and the caudate and their AP level is indicated in section drawings taken at three different rostrocaudal levels shown from caudal to rostral. **B** Summary view of the location of the cortical injection sites mapped onto a drawing of a template hemisphere. For the sake of simplicity, in this view only the injection sites of the cases shown in Figures 5, 6 and 7 are included. The arcuate, intraparietal, and superior temporal sulci are shown as “opened” to better show the location of the injection sites in the banks. C, central sulcus; IA, inferior arcuate sulcus; IP, intraparietal sulcus; L. lateral fissure; Lu, lunate sulcus; P. principal sulcus; SA, superior arcuate sulcus; Spur, spur of the arcuate sulcus; ST, superior temporal sulcus

Once the appropriate site was chosen, the neural tracers Fast Blue (FB, 3% in distilled water, Dr Illing Plastics GmbH, Breuberg, Germany), Diamidino Yellow (DY, 2% in 0.2 M phosphate buffer at pH 7.2, Dr Illing Plastics), True Blue (TB, 5% in distilled water, EMS-POLYLOY GmbH, Gross-Umstadt, Germany), Wheat Germ Agglutinin (WGA; 4% in distilled water, Vector Laboratories, Burlingame, CA), Dextran conjugated with Lucifer Yellow (Lucifer Yellow Dextran, LYD, 10 000 MW, 10% 0.1 M phosphate buffer, pH 7.4; Invitrogen, Thermo Fisher Scientific, Waltham, MA) or with tetramethylrhodamine (Fluoro-Ruby, FR, 10% 0.1 M phosphate buffer, pH 7.4; Invitrogen), Cholera Toxin B subunit, gold conjugated (CTB gold, 0.5% in distilled water, LIST, Campbell, California), or conjugated with Alexa 488 (CTB green) or Alexa 594 (CTB red; 1% in 0.01 M phosphate-buffered saline at pH 7.4, Molecular Probes, Thermo Fisher Scientific), were slowly pressure-injected in the cases of striatal injections through a stainless steel 31 gauge beveled needle attached through a polyethylene tube to a Hamilton syringe (Hamilton Company, Reno NV), or in the cases of cortical injections through a glass micropipette (tip diameter: 50–100 μm) attached to a 5- or 10-μL Hamilton micro syringe, positioned with a stereotaxic holder. For all tracer injections in the striatum, the needle was lowered within a guiding tube to avoid tracer spillover in the white matter. Table 1 and Fig. 1 summarize the locations of the injections, the injected tracers, and the amounts injected.

After appropriate survival periods following the injections (48 h for WGA, 15–28 days for the other tracers), each animal was deeply anesthetized with an overdose of sodium thiopental and perfused through the left cardiac ventricle consecutively with saline (about 2 L in 10 min), 3.5% formaldehyde (5 L in 30 min), and 5% glycerol (3 L in 20 min), all prepared in 0.1 M phosphate buffer, pH 7.4. Each brain was then blocked coronally on a stereotaxic apparatus, removed from the skull, photographed, and placed in 10% buffered glycerol for 3 days and 20% buffered glycerol for 4 days. Finally, each brain was cut frozen into coronal sections of 60-μm or 50-μm (Cases 62, 71, 76 and 77) thickness.

In all cases in which fluorescent neural tracer were injected (FB, DY, TB, CTB-green, CTB-red), sections spaced 300 μm apart—that is one section in each repeating series of 6 in Cases 62, 71, 76, and 77 and one in series of 5 in the other cases—were mounted, air-dried, and quickly coverslipped for fluorescence microscopy. Other series of sections spaced 300 μm apart were processed for visualizing CTB green (Cases 64, 75, and 77), LYD (Cases 58 and 62), FR (Case 62) or WGA (Cases 39, 42, 76, and 77) with immunohistochemistry. As in all these cases, an additional injection of the (mostly) anterograde neural tracer biotinylated dextran-amine (BDA) was placed at the cortical level but not considered for the present study, to distinguish the BDA-labeling from the labeling of the other neural tracers visualized using biotinylated secondary antibodies, the sections were processed for the visualization of both BDA and FR, LYD, CTB green, or WGA, using the double labeling protocol described in detail in Gerbella et al. (2010, 2016). Briefly, the sections were first processed to visualize BDA, i.e., incubated overnight in the ABC solution (Vectastain ABC kit, PK-4000, Vector Laboratories) and then BDA was stained brown using 3,3′-diaminobenzidine (DAB, Sigma-Aldrich, St. Louis, MO). Then, the sections were incubated overnight in avidin–biotin blocking reagent (SP-2001, Vector Laboratories), and for 72 h at 4 °C in a primary antibody solution of rabbit anti-FR, or rabbit anti-LY (1:3000; Invitrogen), or rabbit anti-Alexa 488 (1:15,000, Thermo Fisher Scientific) in 0.5% Triton, 5% normal goat serum (Vector Laboratories) in PBS, or overnight at room temperature in a primary antibody solution of goat anti-WGA (1:2000; Vector Laboratories) in 0.3% Triton and 5% normal rabbit serum (Vector Laboratories) in PBS. The sections were then incubated for 1 h in biotinylated secondary antibody (1:200, Vector Laboratories) in 0.3% Triton, 5% normal goat serum (normal rabbit serum for WGA) in PBS. Finally, FR, LYD, CTB green, and WGA labeling was visualized using the Vectastain ABC kit and the Vector SG peroxidase substrate kit (SK-4700, Vector Laboratories) as a chromogen. With this procedure, BDA labeling was stained brown and the FR, LYD, or CTB green labeling was stained blue in the same tissue sections. In Case 30, CTB-green labeling was visualized only in fluorescence microscopy. In Case 20, CTB gold was revealed by the silver-intensification protocol described by Kritzer and Goldman-Rakic (1995).

In all cases, sections spaced 300 μm apart were stained with the Nissl method (0.1% thionin in 0.1 M acetate buffer, pH 3.7).

### Data analysis

**Fig. 2:**
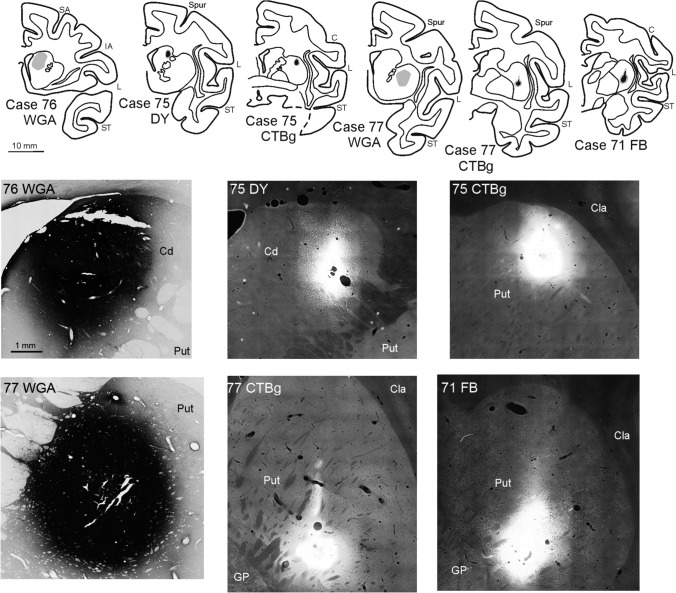
Location of the injection sites in the striatum shown in drawings of coronal sections and in brightfield (for WGA injections) and epifluorescence (for CTB green, DY, and FB injections) photomicrographs. In the drawings, all injection sites except for WGA are depicted as a black zone corresponding to the core, surrounded by a gray zone, corresponding to the halo. WGA injection sites are depicted as a gray zone because of the poor definition of the core versus the halo. Calibration bars shown for the section drawing and the photomicrograph of Case 76 WGA apply to all section drawings and photomicrographs, respectively. Cd, caudate nucleus; Cla, claustrum; CTBg, CTB green; GP, globus pallidus. Other abbreviations as in Fig. 1

All the injection sites used in this study were completely confined to the target striatal nucleus (caudate or putamen) or to the thickness of cortical areas. Photomicrographs of the striatal tracer injections used in the present study are shown in Fig. 2. For the areal attribution of the injection sites and of the labeled corticostriatal neurons, the cortex was subdivided according to architectonic or connectional criteria described in detail in Caminiti et al. (2017).

In the cases of striatal injections, the distribution of the corticostriatal labeled neurons was analyzed as described in detail in Borra et al. (2022). In all cases, the distribution of retrograde labeling in the ipsilateral claustrum was plotted in sections every 600 μm together with the claustral border, using a computer-based charting system. Data from individual sections were then imported into the 3-dimensional (3D) reconstruction software (Demelio et al. 2001) providing volumetric reconstructions of the monkey claustrum. This procedure is the same used for the visualization of the distribution of corticostriatal neurons in 3D reconstructions of the ipsilateral hemisphere. In most cases, sections through the contralateral claustrum have been examined to look for retrograde and/or anterograde labeling. This labeling was absent after the striatal neural tracer injections and negligible after the cortical injections.

In the cases in which the total number of cortical neurons labeled after striatal or cortical tracer injections was available, the weight of claustrostriatal and claustrocortical projections was expressed as percentage of labeled neurons found in the claustrum, relative to the overall cortical labeling.

**Fig. 3:**
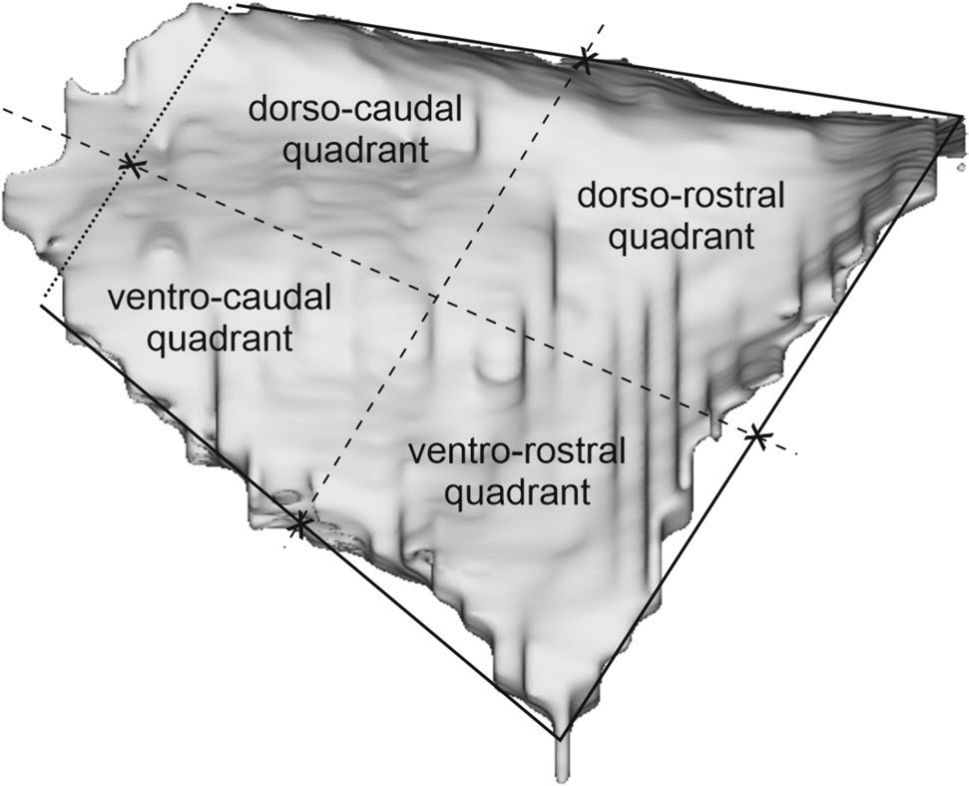
Subdivision of the claustrum into quadrants. A medial view of a 3D claustral reconstruction was inscribed into a trapezoid in which 3 sides are represented by the dorsal, anterior, and ventral border of the claustrum. The fourth side is parallel to the anterior one and placed in the caudal part of the 3D reconstruction, just rostral to its rounded end. The two lines connecting the middle of opposite sides define the quadrants

To describe the location of the claustral labeling, the medial views of the 3D claustral reconstructions were subdivided into quadrants, similarly to Gamberini et al. (2017, 2021), as shown in Fig. 3. Specifically, each 3D reconstruction was inscribed into a trapezoid in which 3 sides were represented by the dorsal, anterior, and ventral border of the claustrum. The fourth side was parallel to the anterior one and placed in the caudal part of the 3D reconstruction just rostral to its rounded end. Then, two lines connecting the middle of opposite sides defined the quadrants. The claustral labeling was then analyzed in terms of percent distribution of the labeled cells in each quadrant.

Furthermore, composite views of the overall distribution of the claustral labeled regions observed after all the neural tracer injections were obtained by warping with Adobe Photoshop individual medial views of claustral reconstructions to a template one (Case 77 right) using as reference points the geometrical landmarks adopted for defining claustral quadrants (Fig. 3). As the reconstructions of different cases were very similar, the distortions caused by this warping procedure were in general small. Claustral labeled territories were then delineated by excluding scattered labeled neurons. For areas injected in more than one case, the labeled territory was defined by combining individual labeling distributions.

## Results

### Claustrostriatal projections

**Fig. 4:**
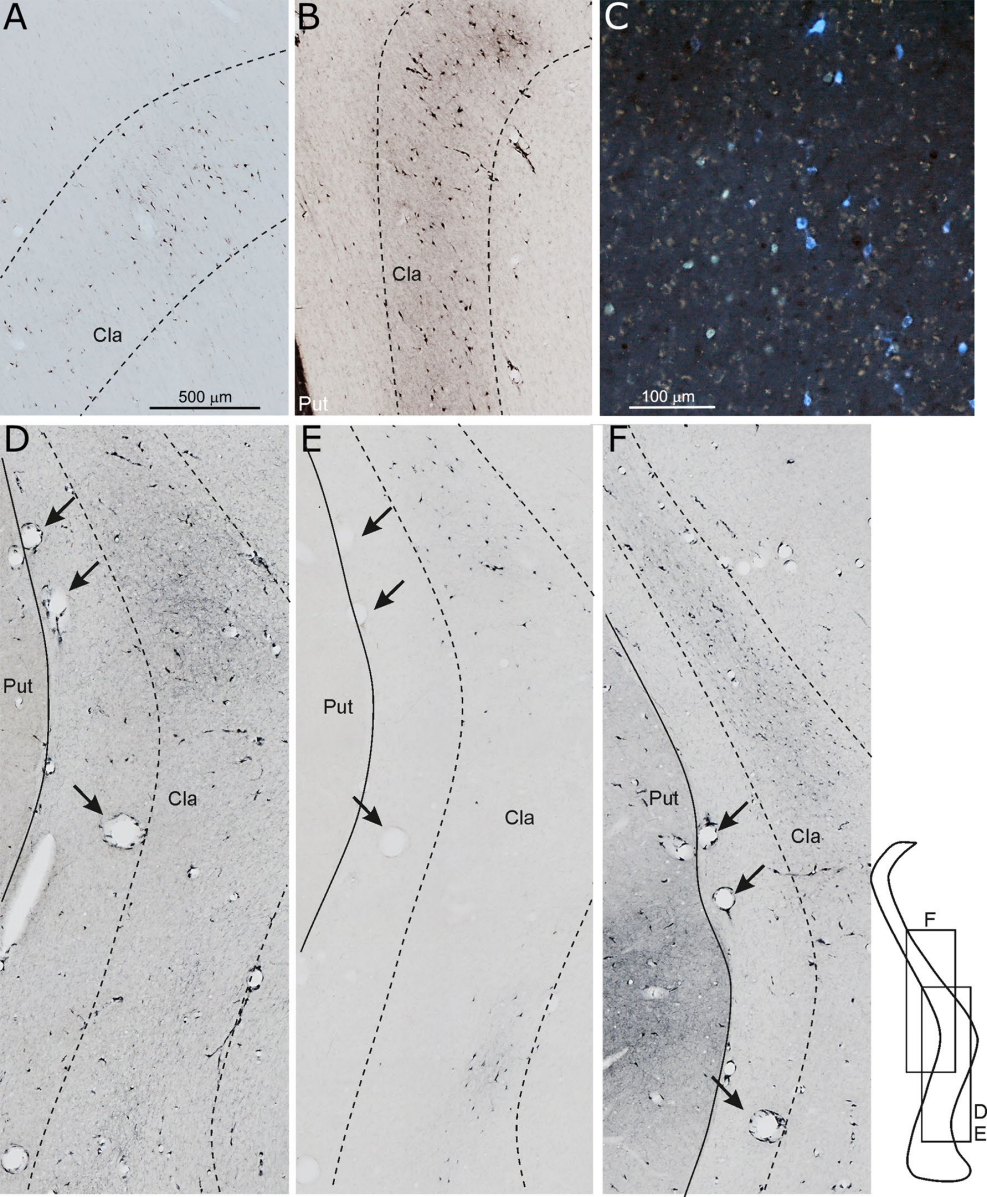
Low-power photomicrographs showing examples of retrograde and anterograde labeling observed in the claustrum. **A**, **B** Claustral neurons labeled after tracer injections in the caudate (Case 76 WGA) and in the putamen (Case 75 CTB green), respectively. **C** DY (yellow-green) and FB (light blue) labeled claustral neurons observed after injections in areas 8-FEF and 45B, respectively, in Case 36. D-F: photomicrographs from adjacent sections through the claustrum taken from Case 62 showing retro-anterograde labeling observed after LYD injection in parietal area PFG and AIP (D), CTB green injection in the intermediate part of prefrontal areas 46v and 12r (**E**), and FR injection in the hand field of F1 (**F**). Arrows in (**D**–**F**) point to the same blood vessels. Scale bar shown in (**A**) applies also to (**B**, **D**, **E**, and **F**)

In all the cases of tracer injections in the striatum, relatively rich retrograde labeling was observed in the claustrum, where the labeled cells were rather homogeneously distributed over relatively large claustral zones (Fig. 4A, B). The claustral labeled cells were about 2% (1–3%) of the total number of neurons labeled in the ipsilateral cerebral cortex by the same injections. This percentage of labeled cells was comparable to that observed for several cortical areas (Borra et al. 2022). The location of the labeled claustral regions, however, varied according to the location of the tracer injection in the striatum.

**Fig. 5:**
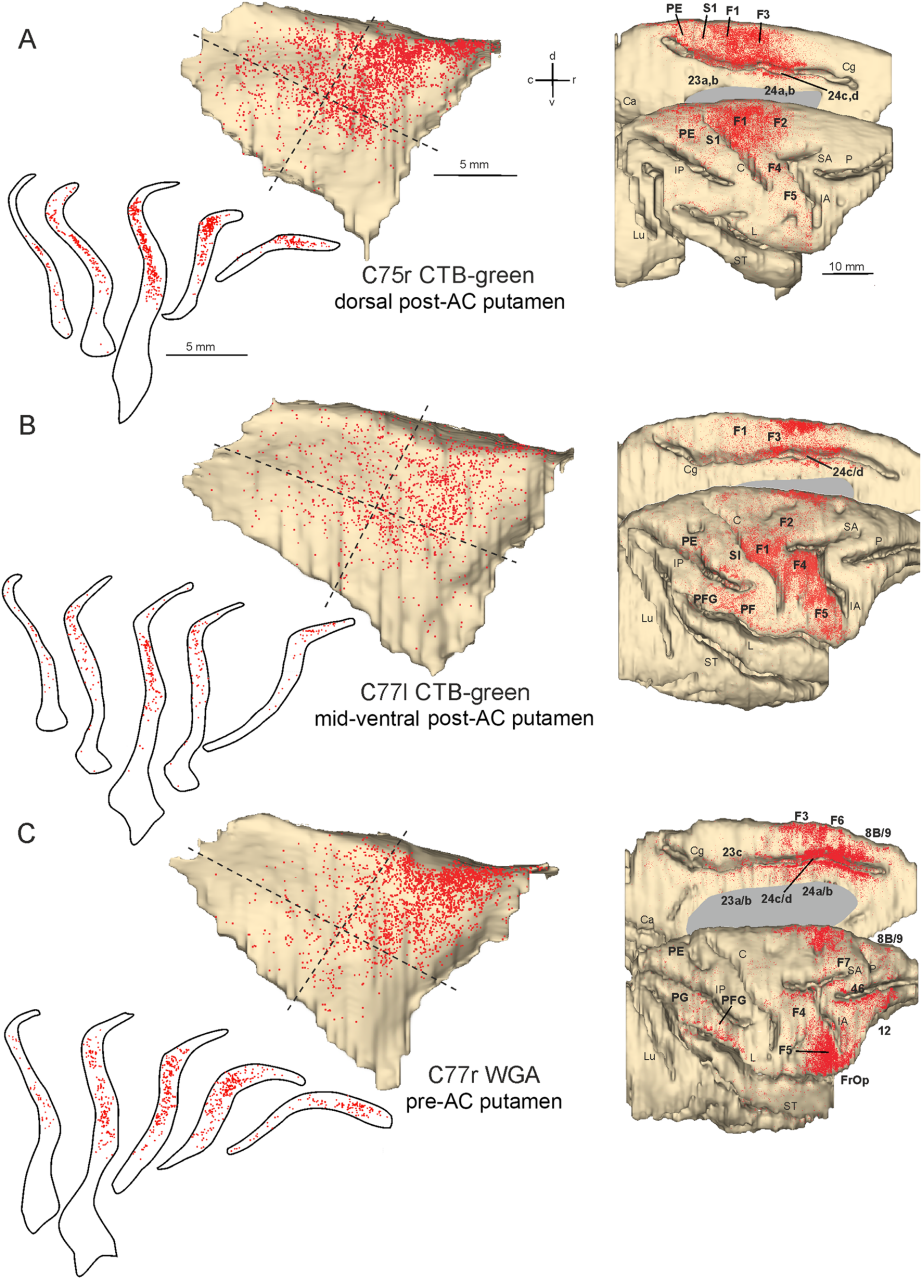
Distribution of the claustral and cortical labeling observed after the tracer injections placed in the putamen at different rostrocaudal and dorsomedial levels in Cases 75r CTB green (**A**), 77 l CTB green (**B**), and 77r WGA (**C**) Each dot represents a labeled neuron. On the left, the claustral labeling is shown on a medial view of the 3D reconstruction of the claustrum and in drawings of coronal sections. On the 3D reconstructions, dashed lines define the quadrants. On the right, the cortical labeling is shown on dorsolateral and medial views of the 3D reconstruction of the injected hemisphere. For the sake of comparison, in this and in subsequent figures all claustral reconstructions and sections are shown as left and to keep the same rostrocaudal orientation, all the hemispheres as shown as right. Scale bars shown in A apply also to (**B**, **C**). C, central sulcus; Ca, calcarine fissure; Cg, cingulate sulcus; FrOp, frontal opercular area; IA, inferior arcuate sulcus; IP, intraparietal sulcus; L, lateral fissure; Lu, lunate sulcus; P, principal sulcus; SA, superior arcuate sulcus; Spur, spur of the arcuate sulcus; ST, superior temporal sulcus

In Cases 75r CTB green and 77 l CTB green (Fig. 5A, B), the injection site involved the post-commissural putamen: in the cortex, most labeling was found in the primary motor area F1 and premotor and cingulate motor areas, to a lesser extent in primary somatosensory area and rostral parietal areas. In both cases the labeling in the claustrum was densest in the caudal two thirds of the dorso-rostral quadrant and extended also more ventrally and caudally at the junction of the four quadrants.

In Case 77r WGA (Fig. 5C), the injection site was placed more rostrally in the pre-commissural putamen and the cortical labeling was densest in rostral premotor areas and ventrolateral prefrontal cortex, weaker in rostral inferior parietal areas. The claustral labeled sector was more confined to the dorso-rostral quadrant, tending to extend more rostrally than that observed after the tracer injections in the post-commissural putamen.

**Fig. 6:**
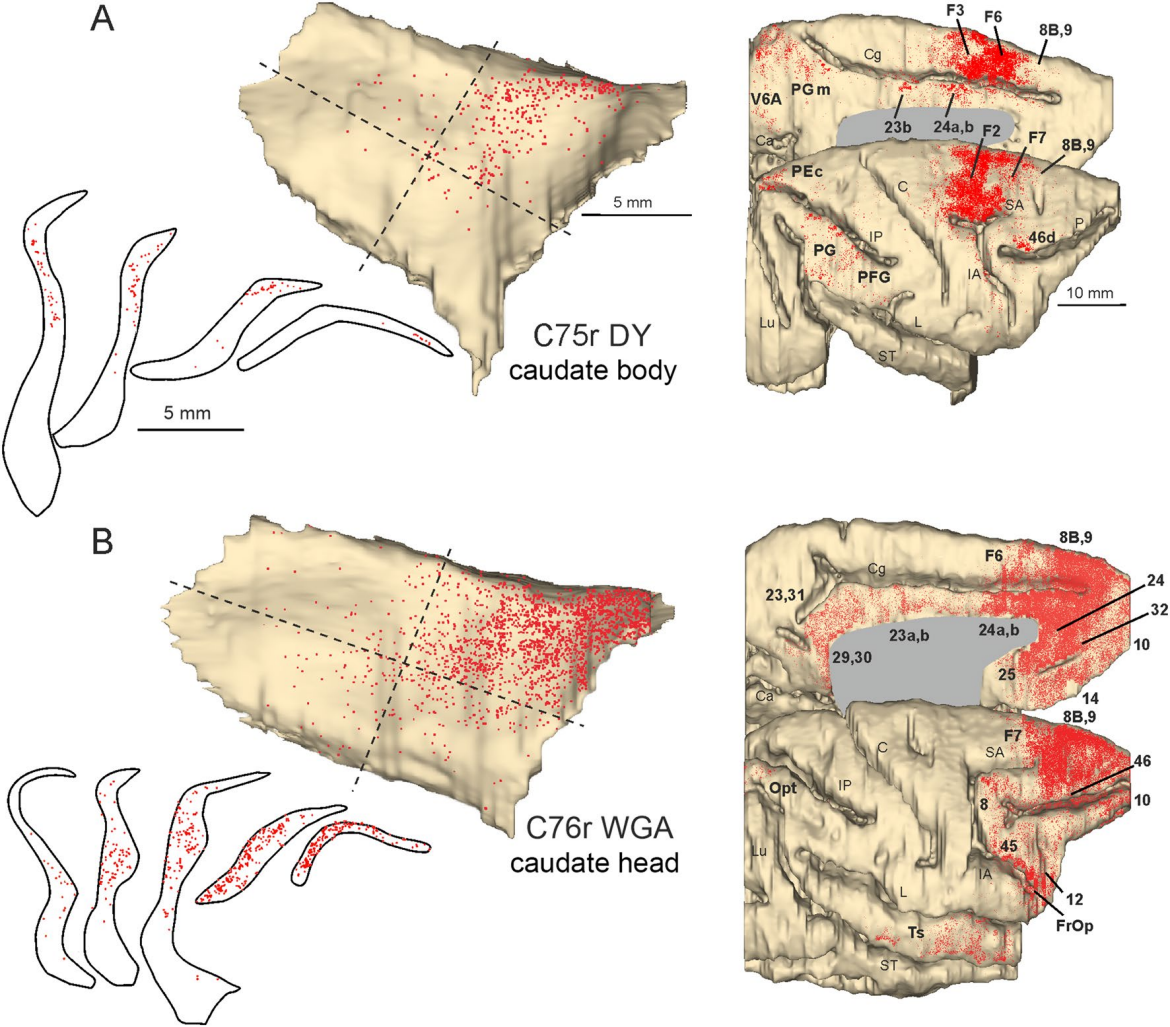
Distribution of the claustral and cortical labeling observed after the tracer injections placed in the caudate body in Cases 75r DY (**A**) and in the caudate head in Case 76r WGA (**B**) Conventions and abbreviations as in Fig. 3

In Case 75r DY (Fig. 6A), the injection site involved a caudate sector recipient of projections originating mostly from dorsal and medial premotor areas, but also from caudal superior and inferior parietal areas. The claustral labeled cells were virtually all located in the dorso-rostral quadrant, overlapping caudally with the claustral sector projecting to the post-commissural putamen.

In Case 76r WGA (Fig. 6B), the tracer injection was placed in a caudate head sector recipient of projections mostly from medial and dorsolateral prefrontal areas and to a lesser extent from caudal ventrolateral prefrontal, caudal cingulate, and rostral superior temporal areas. In the claustrum, the labeling was densest in the rostral two thirds of the dorso-rostral quadrant, thus extending more rostral than the previous case.

Accordingly, both the studied putaminal and caudate regions received the densest projections from the dorso-rostral quadrant of the claustrum, although additional projections to the post-commissural putamen originated also from the anterior part of the dorso-caudal quadrant and the dorsal part of the two ventral quadrants.

### Claustrocortical projections

#### Claustral projections to frontal skeletomotor and oculomotor areas

**Fig. 7:**
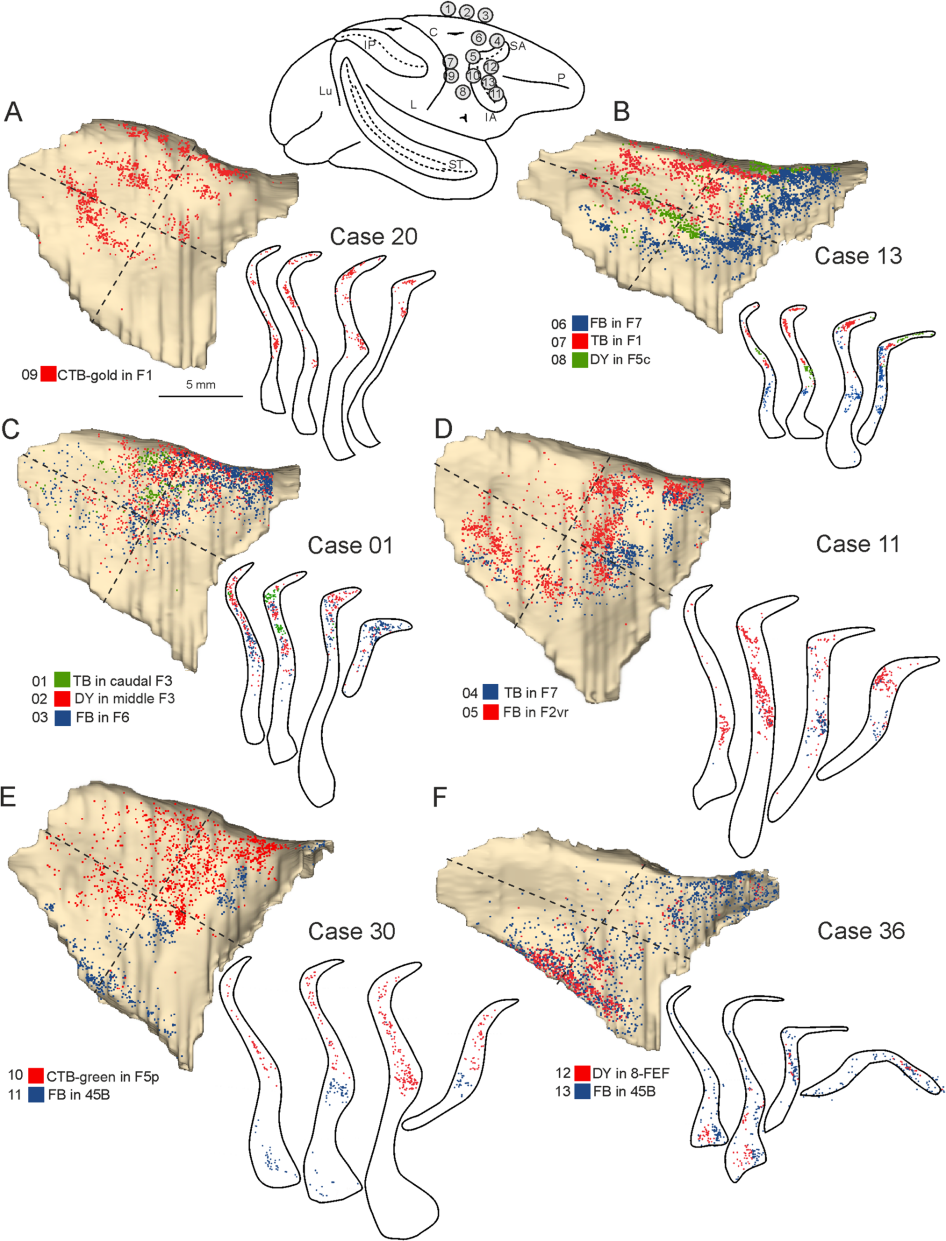
Distribution of the claustral labeling observed after the tracer injections placed in the caudal frontal skeletomotor and oculomotor areas. On the top, a composite view shows the location of the injection sites. Conventions and abbreviations as in Fig. 3

After the tracer injections in frontal motor areas the distribution of the labeling in the claustrum was not uniform and tended to be organized into distinct clusters forming bands whose location varied according to the injected area. The proportion of claustral labeled cells varied across cases but was on average about 7% of the total number of cortical labeled neurons, which is comparable to the main input from connected parietal and prefrontal areas. Specifically, projections to the primary motor area F1, as observed in Cases 20 l CTB-gold and 13 TB (Fig. 7A, B), originated mainly from the dorso-caudal quadrant of the claustrum, but also from the caudal half of the dorso-rostral and the dorsal part of the ventro-caudal quadrant. In both cases the labeled cells formed distinct bands running in rostro-caudal direction. Projections to supplementary motor area F3 (Fig. 7C) originated from a claustral sector straddling the border between the two dorsal quadrants; those to premotor area F5 (F5p and F5c, Fig. 7B, E) from the caudal half of the dorso-rostral quadrant and from the dorsal part of the ventro-caudal quadrant. Projections to area F2 (F2vr; Fig. 7D) involved a dorso-rostral sector largely overlapping that projecting to the other caudal frontal motor areas and a sector of the ventro-caudal quadrant located more ventral to that projecting to F1 and ventral premotor area F5. Projections to pre-supplementary areas F6 and dorso-rostral premotor area F7 (Fig. 7B–D) originated from the dorso-rostral quadrant also including the rostral part of it and for F7 also from the two ventral quadrants. Projections to oculomotor areas 8-FEF (Frontal Eye Field) and 45B originated from the ventral part of the claustrum straddling the two ventral quadrants and, for area 45B, also from the rostral part of the dorso-rostral quadrant. In the ventral claustrum, the cells projecting to area 8-FEF and 45B tended to be segregated more medially and more laterally, respectively (Fig. 4C).

#### Claustral projections to prefrontal areas

**Fig. 8:**
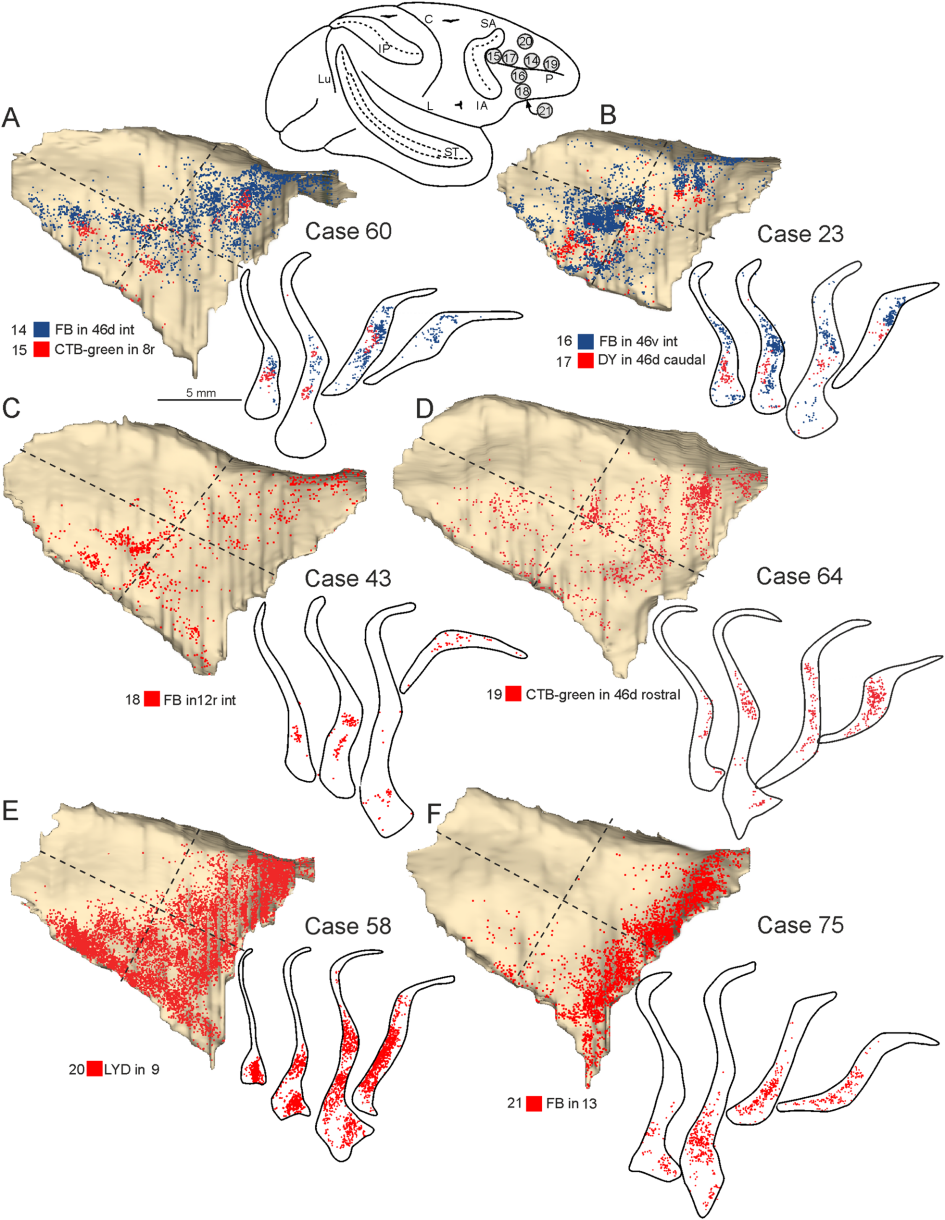
Distribution of the claustral labeling observed after the tracer injections placed in prefrontal areas. On the top, a composite view shows the location of the injection sites. Conventions and abbreviations as in Fig. 3

As for frontal motor areas, the proportion of claustral labeled cells projecting to the prefrontal areas varied across cases but was on average about 7% of the total number of cortical labeled neurons. After the tracer injections in the lateral part of the prefrontal cortex labeled cells involved a large claustral sector running from the dorso-rostral to the ventro-caudal quadrant (Fig. 8A–D). However, projections to intermediate and rostral sectors of area 46d were stronger from the dorso-rostral quadrant, whereas those to intermediate 46v and 12r from the ventro-caudal one. In the cases with two tracer injections in different prefrontal areas (Fig. 8A, B), labeled cells, though involving the same claustral sector, tended to be organized into distinct clusters. In Case 58 LYD (area 9, Fig. 8E) the labeling was extremely dense at both the cortical and subcortical level. In the claustrum it involved a large sector including the two anterior quadrants and the ventral part of the ventro-caudal one. Finally, the tracer injection placed in orbitofrontal area 13 (Fig. 8F) labeled a territory running along the rostral border of the claustrum.

#### Claustral projections to parietal and other cortical areas

**Fig. 9:**
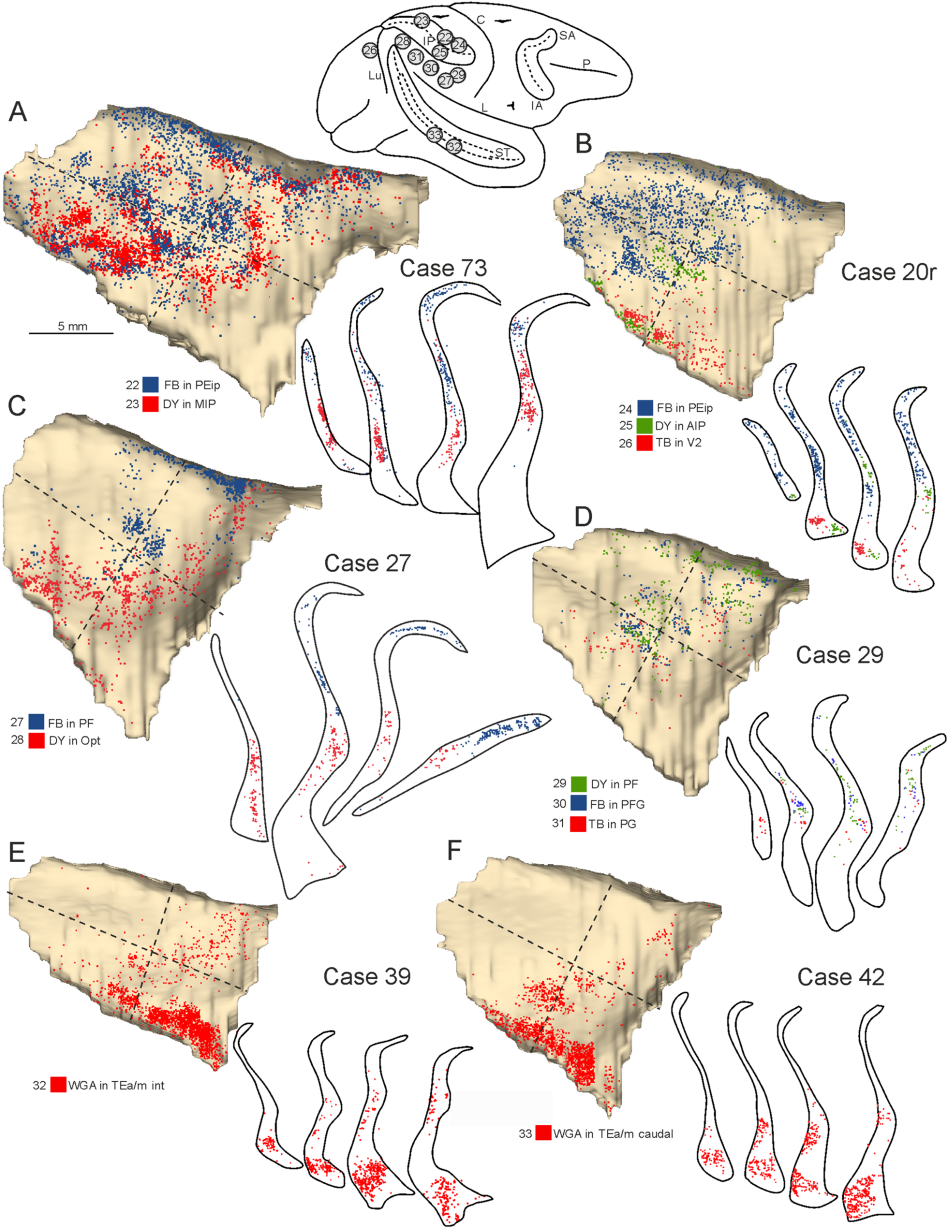
Distribution of the claustral labeling observed after the tracer injections placed in the parietal, occipital, and temporal areas. On the top, a composite view shows the location of the injection sites. Conventions and abbreviations as in Fig. 3

After the tracer injections in parietal areas, the proportion of claustral labeled cells was about 6% of the total number of cortical labeled neurons, thus comparable to that observed after the tracer injections in frontal motor and prefrontal areas. As observed for frontal motor areas, labeled cells tended to aggregate in bands or clusters. Specifically, cells projecting to intraparietal area PEip (Fig. 9A, B) occupied the dorso-caudal quadrant extending also to the ventro-caudal and, more weakly, to the dorso-rostral one. In contrast, cells projecting to medial intraparietal area MIP (Fig. 9A) were mostly located in the ventro-caudal quadrant with some clusters dispersed in the other quadrants. After the tracer injections in the inferior parietal lobule, claustral cells projecting to rostral areas PF, PFG, and anterior intraparietal area AIP tended to be more concentrated at the intersection of the four quadrants, also involving more sparsely the dorso-rostral one (Fig. 9B–D). In addition, labeled cells projecting to AIP were also observed close to the ventral border of the claustrum. In contrast, claustral cells projecting to caudal areas PG and Opt tended to be located more ventrally and rostrally to those projecting to more rostral areas (Fig. 9C, D).

Furthermore, to provide a more general view of the topography of the claustrocortical projections, we analyzed the distribution of the claustral labeling after two neural tracer injections in the inferotemporal area TEa/m and one in visual area V2. In the first case, the proportion of claustral labeled cells was about 8% of the total number of cortical labeled neurons and the labeling very densely involved the ventral part of the claustrum, with additional clusters forming a band running obliquely in its rostral sectors (Fig. 9E, F). The claustral projections to V2 involved the ventral part of the claustrum, which was labeled also after the tracer injection in AIP, but V2- and AIP-projecting neurons were clearly segregated in the mediolateral extent (Fig. 9B). A recent study suggests that in the ventral part of the claustrum there exists a sector where neurons are generated at an earlier stage during embryonic development relative to the rest of the claustrum, which might represent a basis for differential circuits formation (Li et al. 2023).

### Topography of claustrostriatal and claustrocortical projections*.*

**Fig. 10:**
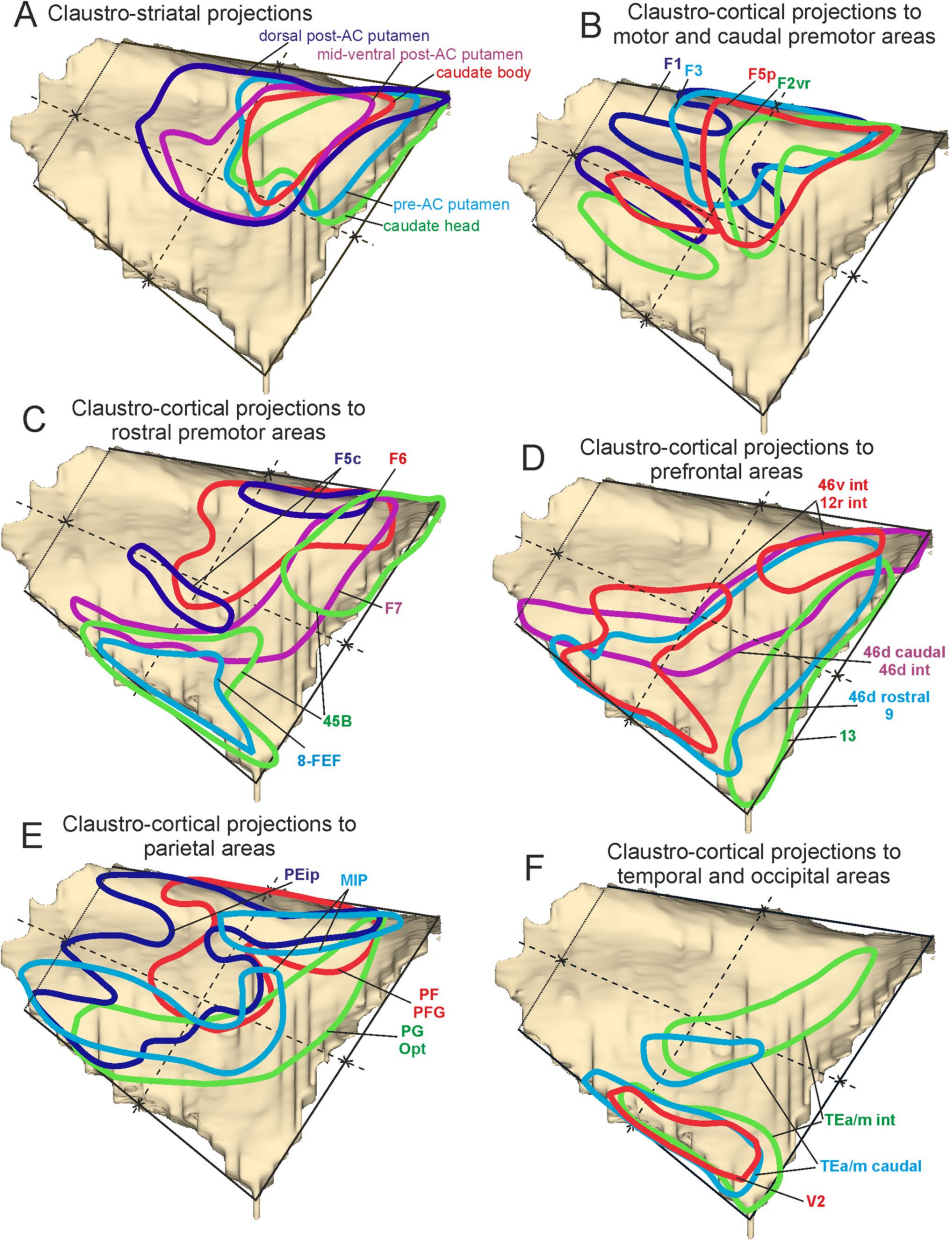
Composite views of the claustral territories labeled after the tracer injections placed in the various striatal sectors (**A**) and cortical areas (**B**–**F**), warped on a template 3D reconstruction (Case 75r)

**Fig. 11:**
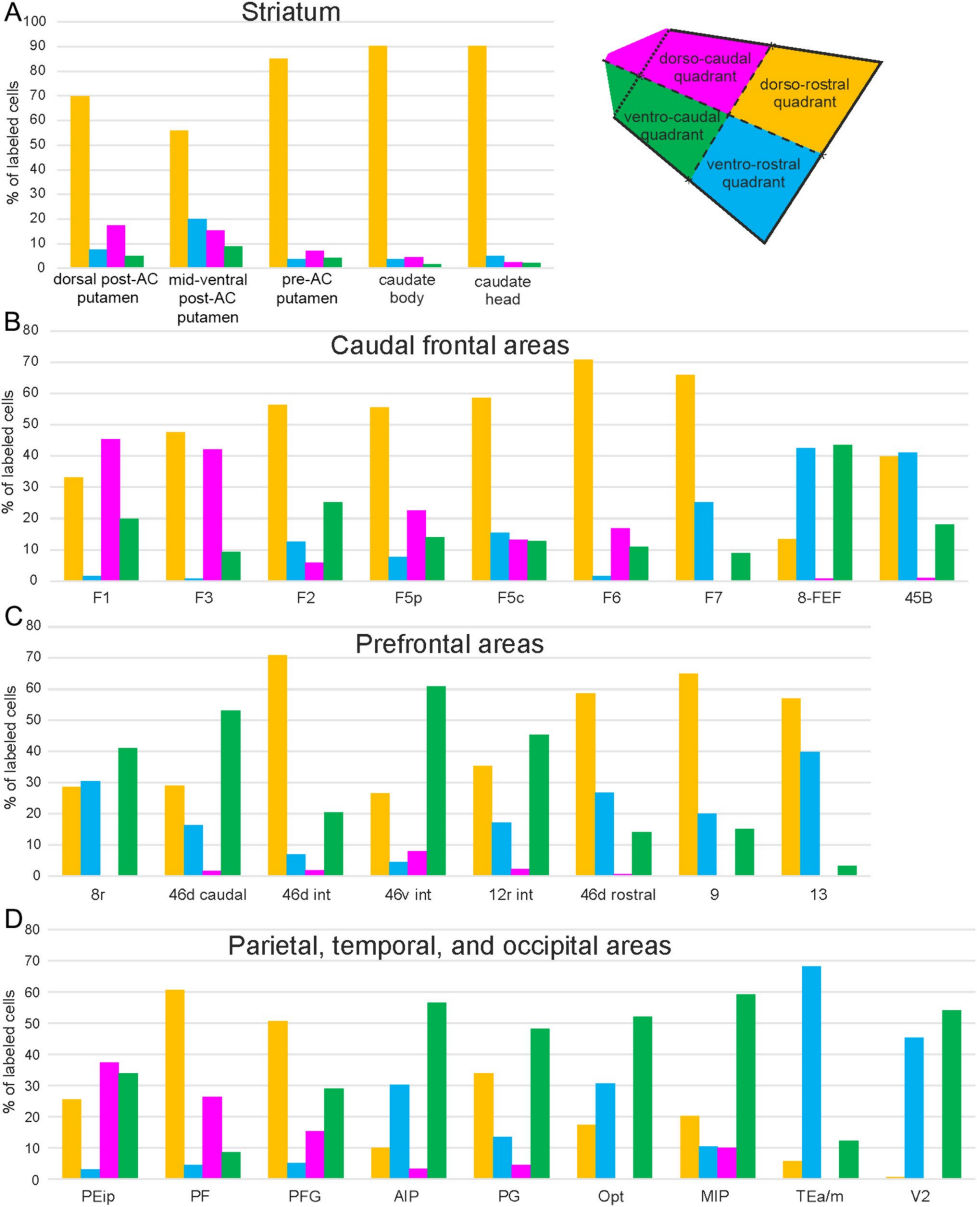
Percent distribution of the retrograde labeling observed in the different quadrants of the claustrum (top right) after the tracer injections placed in different parts of the striatum and in prefrontal, caudal frontal, parietal, temporal, and occipital areas. All the cases of retrograde tracer injections listed in Table 1 were used for this analysis. For the areas injected in more than one case, the bar graphs show the mean values

The next step of the analysis had two main goals: i) to estimate the degree of overlap of the claustral zones labeled after the tracer injections in the striatum or in the various cortical areas; and ii) to determine whether areas or striatal sectors involved in the same network receive information from overlapping claustral zones. To these aims, individual 3D claustral reconstructions were warped to a common template (Fig. 10). In premise, it should be noted that the absolute number of claustral labeled neurons varied to some extent across different cases, which can be accounted for by the differences in amount, spread, and sensitivity between different tracer injections. This variability could be reflected in the dimension of the labeled zone but much less in its relative position. In this analysis the position of the labeled zone was defined based on the distribution of the whole labeling, excluding only few scattered neurons. Furthermore, the distribution of the labeling in the various quadrants was analyzed in terms of percent distribution thus was normalized across cases (Fig. 11). In general, the results showed an extensive overlap of claustral zones projecting to either different striatal territories or different cortical areas, reflecting a relatively widespread nature of claustral connectivity. Specifically, warping of claustral zones labeled after the tracer injections in the striatum shows overlap but also a rostrocaudal segregation of the labeled zones (Fig. 10A), together with a clear dominance of the dorso-rostral quadrant (Fig. 11A) when the injection site moved from the post-commissural to pre-commissural putamen and to the caudate head. Furthermore, claustral zones labeled after tracer injections in a specific region show extensive overlap for some areas, but also a clear segregation for other areas (Fig. 4D-F). As an example, overlapping zones projected to F1 and caudal premotor areas F3, F2vr, and F5p, but segregation existed for those projecting to F7 and, in part, to F2vr (Fig. 10B, C). Figure 11B shows a tendency to an increasing contribution of the dorso-rostral quadrant moving from F1 and F3 to more rostral frontal areas and a predominance of the dorso-caudal quadrant in the projections to F1, F3, and F5p, which was very low and absent for F2vr and F7, respectively. Furthermore, projections to F7 were characterized by an increased contribution of the ventro-rostral quadrant. As for the prefrontal cortex, there was a clear segregation of the zones projecting to orbitofrontal area 13 and to areas 46v and 46d and a clear overlap of both these zones with that projecting to area 9 (Fig. 10D). Figure 11C shows a similar distribution of cell labeling for areas 8r and caudal 46d, where the main contribution originated from the ventro-caudal quadrant, which was also the main source of projections to intermediate areas 46v and 12r. In contrast, the main contribution to intermediate and rostral area 46d and areas 9 and 13 originated from the dorso-rostral quadrant. As for the parietal cortex, the claustral zones projecting to the superior parietal areas PEip and MIP were only partially overlapped, while those projecting to the inferior parietal areas PF and PFG and those projecting to PG and Opt were almost completely segregated (Fig. 10E). Figure 11D shows a relatively strong contribution of the dorso-caudal quadrant to somatomotor areas PEip and PF, whereas the main contribution to the caudal visuomotor areas PG, Opt, and MIP was from the ventro-caudal quadrant.

**Fig. 12:**
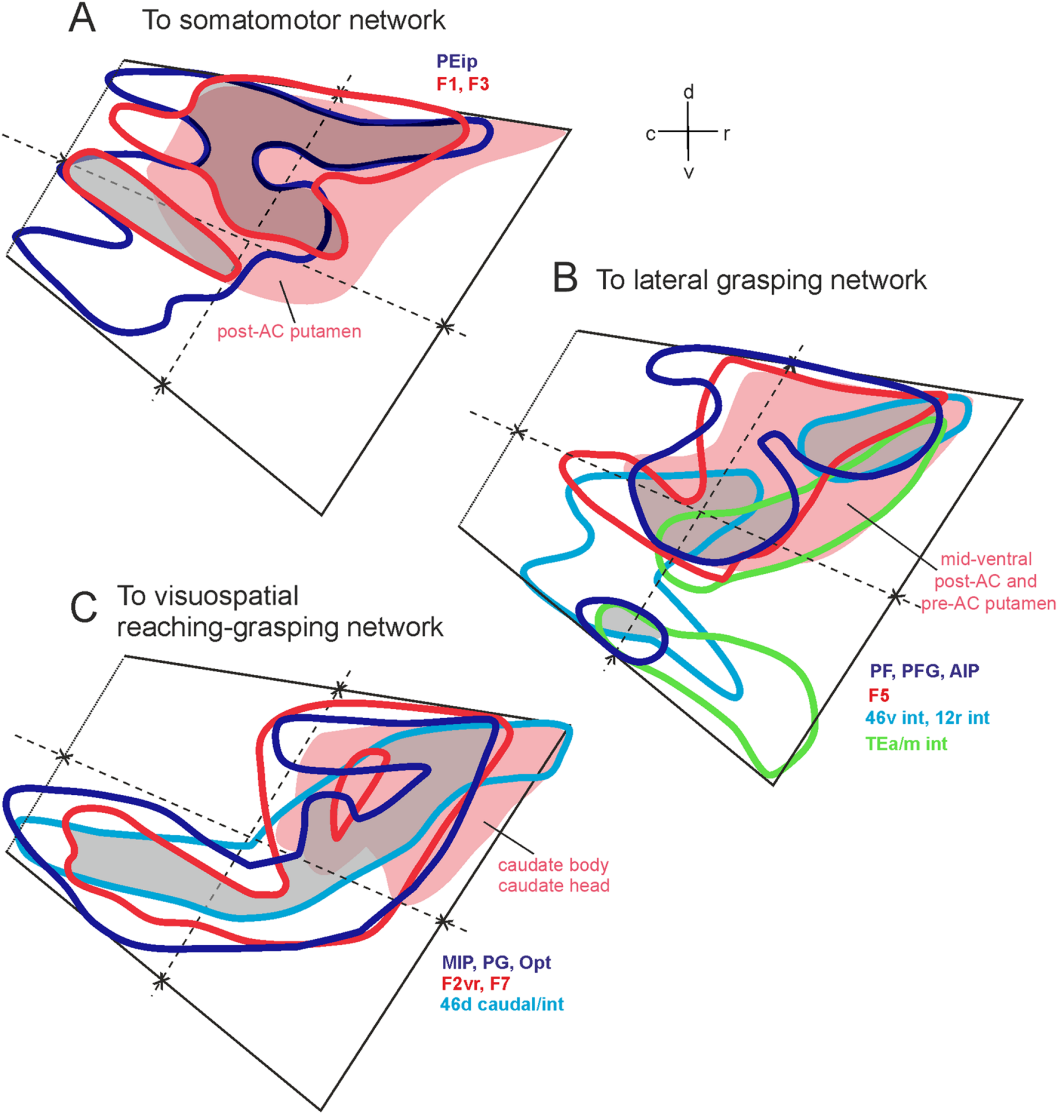
Composite views of the claustral territories projecting to the prefrontal (light blue lines), caudal frontal (red lines), parietal (blue lines), and temporal (green lines) areas of the somatomotor (**A**), lateral grasping (**B**), and visuospatial reaching-grasping (**C**) network. In each panel, the pink shaded area corresponds to the claustral sector projecting to the striatal zone target of the areas of the network. In each panel, grey shaded areas highlight overlap of two (**A**) or three (**C**, **D**) claustral territories projecting to cortical regions. For the sake of simplicity, the claustral 3D reconstruction has been removed and the structure is schematically represented by the trapezoid perimeter subdivided in quadrants

Figure 12 shows that the degree of overlap markedly increased when projections to areas of the same functional domain or network were compared. Specifically, Fig. 12A shows a claustral sector straddling over the two dorsal quadrants where zones projecting to interconnected somatomotor superior parietal, primary motor, and premotor areas (Caminiti et al. 2017) largely overlap. This sector is also coextensive with that projecting to the post-commissural putamen which, in turn, was target primarily of projections originating from primary motor, caudal premotor, and rostral parietal areas. Figure 12B shows two more dorsal and one very ventral claustral sectors where zones projecting to interconnected parietal, premotor, prefrontal, and temporal areas of the lateral grasping network (Borra et al. 2017) overlap. The two more dorsal zones were included in the larger claustral sector projecting to mid-ventral post-commissural and pre-commissural putamen which, in turn, was target of parietal, premotor, and prefrontal hand-related areas. Figure 4D, E show overlap of retro-anterograde labeling in a mid-dorsal and in a more ventral claustral zone observed in adjacent sections of Case 62 after the injections of LYD in inferior parietal areas PFG and AIP (D) and CTB-green in the intermediate part of prefrontal areas 46v and 12r (E). Figure 4F shows more dorsal FR retro-anterograde labeling observed in a further adjacent section after the injection in the hand field of area F1. Finally, Fig. 12C shows a claustral zone running obliquely from the dorso-rostral to the caudo-ventral quadrant projecting to interconnected areas involved in visuospatial control of reaching and grasping movements (areas MIP, Opt and PG, F2vr, F7, and 46d). In the dorso-rostral quadrant these claustral efferent zones overlap with that projecting to the caudate sector, which was target of projections from dorsal prefrontal, dorsal premotor, and caudal superior and inferior parietal areas.

## Discussion

This study is the first in addressing the issue of the organization of the claustrostriatal projections in macaques, further highlighting the complexity of information processing of the basal ganglia in motor and non-motor functions. Such projections show a rough topography, since a claustral efferent zone projects to both a specific striatal territory and to cortical areas involved in a network projecting to the same striatal zone. Accordingly, the claustrum would exert its influence on both cortical and cortico-basal ganglia circuits.

### Claustral connectivity

A claustrostriatal projection in the non-human primate has been reported by Arikuni and Kubota (1985) and noted by Griggs et al. (2017) and Borra et al. (2020) but it has never been described in detail or considered in functional models of claustral or striatal circuitry (e.g., Jackson et al. 2020; Averbeck and O’Doherthy 2022). This connection has not been so far observed in rodents (see Madden et al. 2022), but there is tractographic evidence for it in the human brain (Milardi et al. 2015). Our data show a relatively robust projection from the claustrum, at least to the post-commissural and pre-commissural putamen and to the caudate head. The observation of Griggs et al. (2017) of a claustral labeling after tracer injections in the caudate tail complements our data, suggesting that the whole primate striatum is target of claustral projections. It still remains to be assessed whether claustrostriatal projections are collaterals of claustrocortical ones. In this respect, it should be noted that in the present study the claustral cells projecting to a given striatal zone were distributed in a rather uniform fashion, whereas those projecting to a given cortical area tended to cluster.

Many studies have described in macaques the claustral projections to frontal (Pearson et al. 1982; Tanné-Gariépy et al. 2002; Reser et al. 2014), parietal (Mesulam et al. 1977; Pearson et al. 1982; Minciacchi et al. 1991; Baizer et al. 1993; Leichnetz 2001; Gamberini et al. 2017; 2021) temporal (Pearson et al. 1982; Insausti et al. 1987; Baizer et al. 1993, 1997; Gattass et al. 2014), occipital (Pearson et al. 1982; Kennedy and Bullier 1985), and cingulate (Vogt et al. 1979) areas. Some of them have provided evidence for overlap of claustral zones projecting to different parietal (Gamberini et al. 2017, 2021) and prefrontal (Reser et al. 2014) areas, or to premotor and prefrontal areas (Tanné-Gariépy et al. 2002), ventral premotor and inferior parietal areas (Bruni et al. 2018), and dorsal premotor and rostral somatosensory parietal areas (Pearson et al. 1982). The overlap of projections from prefrontal and parietal interconnected regions was observed by Selemon and Goldman-Rakic (1988). Our study, based on comparison of the results from tracer injections in frontal motor, prefrontal, parietal, and temporal areas, largely extends previous studies providing a more comprehensive view of the topography of the claustrocortical projections in the macaque brain. As a whole, our data support proposed models (see e.g., Jackson et al. 2020; Madden et al. 2022) in which a specific claustral zone projects to different cortical areas, even located in different lobes, but interconnected with one another by cortico-cortical connections shaping specific cortical networks and information processing streams (see Caminiti et al. 2017).

Finally, in the context of the interplay between claustrum, cortex, and striatum, it should be noted that cortical areas are a source of projections to both the claustrum and the striatum and that there are layer V neurons which project to both these structures (Parent and Parent 2006). The possibility that claustral neurons project to both cortical and striatal targets remains to be assessed.

### Functional considerations

The functional role of the claustrum remains elusive. In the last 15–20 years the widespread connectivity with the cortex and functional data mostly from rodent studies, have been used to support several functional hypotheses on the claustrocortical interplay. Accordingly, there has been a growing interest in this structure, even more considering the increasing evidence from human studies that the claustrum plays a role in psychiatric and neurological diseases (see Atilgan et al. 2022; Smith et al. 2022).

In general, the extensive overlap of the claustral connectivity with different interconnected cortical areas suggests a role of the claustrum in trans-claustral transfer of cortico-cortical information as suggested by the so-called “replication principle” (Shipp 2003) for thalamic nuclei (e.g., Guillery 1995; Guillery and Sherman 2002; Kastner et al. 2020; Borra et al. 2023). This states that thalamic nuclei project to cortical areas which are cortico-cortically connected. This mechanism can confer flexibility to cortical computations (Battaglia-Mayer and Caminiti 2019).

Based on its extensive cortical connectivity with sensory areas it has been suggested a role of the claustrum in cross-modal transfer of sensory information and integration (Ettlinger and Wilson 1990) and in coordinating and regulating cortical synchrony of neocortical areas for perceptual binding (Crick and Koch 2005; Smythies et al. 2012). Specifically, it is known that claustral neurons project to different classes of inhibitory cortical interneurons, thus generating strong feedforward inhibition in the cortex (Jackson et al. 2020). This might provide a mechanism for the suppression of distracting sensory stimuli. However, this view is challenged primarily by macaque data showing unimodal sensory responses of claustral neurons (Remedios et al. 2010) and by the lack in rodents of sensory responses in anterior claustrum (Chevée et al. 2022), where cells projecting to the primary somatosensory cortex are located.

A further view on claustral functions emphasizes the connectivity with the anterior cingulate cortex and posits a role in salience detection (Remedios et al. 2014) and selective attention (Goll et al. 2015). In this context, the claustrum would integrate limbic-related information with sensory and motor signals, thus acting as a limbic–sensory-motor interface (Smith et al. 2020). However, the connectivity with anterior cingulate cortex only partially involves the claustrum (Baleydier and Mauguiere 1980). Furthermore, there is clear evidence for a role of the claustrum in motor control: in rodents, activity in the anterior claustrum appears to be related to motor planning rather than to sensory information (Chevée et al. 2022) and in macaques claustral cells show non-selective movement-related activity during the execution of different forelimb movements (Shima et al. 1996).

In the context of the role of the claustrum in regulating cortical excitability, in awake mice large-scale slow-wave activity has been observed during sleep and awake rest (Narikiyo et al. 2020), leading to the hypothesis that the claustrum could be involved in the transition from cortical upstate to down states, thus in sleep regulation (Smith et al. 2020). Indeed, sleep disturbances can be observed in patients with claustral lesions (Atilgan et al. 2022). Recent evidence showed that a possible reptilian homolog of the claustrum has a role in the generation of transient sharp-wave ripples in the dorsal ventricular ridge, which are characteristic of slow wave sleep in both the reptilian and the mammalian brain (Norimoto et al. 2020). This control includes interhemispheric sleep regulation (Fenk et al. 2023), which accounts for the different timing of interhemispheric claustral coupling of slow-wave and REM sleep.

Starting from the idea that the claustrum could be involved in cortical network switching (Reser et al. 2014) and could be viewed as a hub connected with areas involved in executive control, Madden et al. (2022) proposed a model in which the prefrontal areas direct the claustrum to flexibly instantiate cortical networks to subserve cognitive control. However, connectivity with prefrontal areas does not involve the whole claustrum.

The present study provides evidence for different, partially overlapping, claustral efferent zones connected with networks of cortical areas belonging to specific functional domains. This connectional architecture is the substrate for a potential general role of the claustrum in coordinating and synchronizing areas of a given network and for cortical network switching, prompted by change of task demands. The presence of claustral neurons projecting both at cortical and striatal levels, if demonstrated, could provide the substrate for a synchronizing role of the claustrum not only on cortical networks but also on the basal ganglia circuits. However, the linkage of different claustral zones with different sets of cortical areas suggests a multiplex role in motor and non-motor functions. Specifically, the claustral zones connected to the somatomotor network could play a role in motor control by suppressing cortical activity before action initiation. Such non-selective mechanism might provide a powerful filter for cortical activity, permitting activity to emerge only in cortical sites receiving the most intensive excitation (Shima et al. 1996). Furthermore, the partially overlapping claustral zones connected to parietal, frontal motor, and prefrontal areas of the lateral grasping and visuospatial reaching-grasping network could be involved in the instantiation of these networks based on signals originating from prefrontal areas (Madden et al. 2022). In this context, it has been suggested that differences in the anatomical and functional organization between various primate species could be related to specializations of the cortical organization for sensory, motor, and cognitive functions (Baizer et al. 2014, 2020).

The present study suggests an influence of the claustrum also on the striatum, which adds further complexity to the information processing of this structure. The striatum is the main input station of the basal ganglia and displays a modular organization in which different striatal territories, in addition to the thalamus and the substantia nigra, are targets of converging input from cortical areas belonging to large-scale functional networks (Gerbella et al. 2016; Choi et al. 2017). Furthermore, the laminar origin of the cortical projections depends on the area and target striatal zones (Borra et al. 2021). Finally, crossed corticostriatal projections exist, whose amount varies according to the target striatal territory (Borra et al. 2022).

It is generally accepted that one major role of the basal ganglia is to combine sensory, motor, motivational, and cognitive signals necessary for selecting the most appropriate motor behavior to reach a goal in specific contexts (Averbeck and O’Doherty 2022).

Accordingly, it is possible that the proposed role of the claustrum in salience detection and in limbic sensory-motor interfacing contributes to the processes of action selection in the striatum. Furthermore, the claustrum might participate in coordinating and synchronizing both at the cortical and striatal level information related to planning and triggering appropriate movements and preventing conflicting ones. Future studies will clarify whether claustrostriatal projections contact selectively a specific class of striatal projecting neurons, thus acting on the direct and/or the indirect pathway, or local interneurons as demonstrated at the cortical level.

## Data Availability

The data that support the findings of this study are available from the corresponding author upon reasonable request.
